# Case report: Unusual and extremely severe lipoprotein X-mediated hypercholesterolemia in extrahepatic pediatric cholestasis

**DOI:** 10.3389/fped.2022.969081

**Published:** 2022-08-04

**Authors:** Rossella Colantuono, Chiara Pavanello, Andrea Pietrobattista, Marta Turri, Paola Francalanci, Marco Spada, Pietro Vajro, Laura Calabresi, Claudia Mandato

**Affiliations:** ^1^Department of Medicine, Surgery and Dentistry “Scuola Medica Salernitana”, University of Salerno, Baronissi, Italy; ^2^Dipartimento di Scienze Farmacologiche e Biomolecolari, Centro E. Grossi Paoletti, Università degli Studi di Milano, Milan, Italy; ^3^Division of Gastroenterology, Hepatology and Nutrition, Bambino Gesù Children's Hospital, IRCCS, Rome, Italy; ^4^Division of Pathology, Bambino Gesù Children's Hospital, IRCCS, Rome, Italy; ^5^Division of Hepatobiliopancreatic Surgery, Liver and Kidney Transplantation, Bambino Gesù Children's Hospital, IRCCS, Rome, Italy

**Keywords:** cholestasis, hypercholesterolemia, lipoprotein X, spontaneous biliary perforation, extra-hepatic

## Abstract

**Background:**

Lipoprotein X (LpX) - mediated extremely severe hyperlipidemia is a possible feature detectable in children with syndromic paucity of intralobular bile ducts (Alagille syndrome) but rarely in other types of intra- and/or extrahepatic infantile cholestasis.

**Case presentation:**

Here we report on a previously well 18-month child admitted for cholestatic jaundice and moderate hepatomegaly. Laboratory tests at entry showed conjugated hyperbilirubinemia, elevated values of serum aminotransferases, gamma-glutamyl transpeptidase (GGT) and bile acids (100 folds upper normal values). Extremely severe and ever-increasing hypercholesterolemia (total cholesterol up to 1,730 mg/dl) prompted an extensive search for causes of high GGT and/or hyperlipidemic cholestasis, including an extensive genetic liver panel (negative) and a liver biopsy showing a picture of obstructive cholangitis, biliary fibrosis, and bile duct proliferation with normal MDR3 protein expression. Results of a lipid study showed elevated values of unesterified cholesterol, phospholipids, and borderline/low apolipoprotein B, and low high-density lipoprotein-cholesterol. Chromatographic analysis of plasma lipoproteins fractions isolated by analytical ultracentrifugation revealed the presence of the anomalous lipoprotein (LpX). Magnetic resonance cholangiopancreatography and percutaneous transhepatic cholangiography showed stenosis of the confluence of the bile ducts with dilation of the intrahepatic biliary tract and failure to visualize the extrahepatic biliary tract. Surgery revealed focal fibroinflammatory stenosis of the left and right bile ducts confluence, treated with resection and bilioenteric anastomosis, followed by the rapid disappearance of LpX, paralleling the normalization of serum lipids, bilirubin, and bile acids, with a progressive reduction of hepatobiliary enzymes.

**Conclusion:**

We have described a unique case of focal non-neoplastic extrahepatic biliary stenosis of uncertain etiology, presenting with unusual extremely high levels of LpX-mediated hypercholesterolemia, a condition which is frequently mistaken for LDL on routine clinical tests.

## Introduction

Lipoprotein X (LpX) is an abnormal unesterified cholesterol- and phospholipid-rich lipoprotein particle regurgitating from bile into the bloodstream. Its presence is associated to a lipoprotein pattern apparently characterized by an increased concentration of low-density lipoprotein (LDL) cholesterol, which results from an unreliable application of Friedewald equation in this condition (1–3). This may consequently delay LpX detection and adequate management. LpX has been reported to be an early marker of either intra or extra-hepatic bile flow reduction, with a high concordance (95% of cases) between its presence and a histology proven cholestasis (4). In pediatrics, patients with biliary atresia tend to have LpX values higher than those with intrahepatic cholestasis except Alagille Syndrome who may present instead extremely severe LpX-mediated hypercholesterolemia (5). Timely recognition of this distinct metabolic condition resistant to standard lipid-lowering drugs is necessary to plan alternative strategies to counteract disfiguring skin xanthomas, blood hyper viscosity-related pulmonary embolism (6) and the (still debated) risk of cardiovascular complications (7).

Here we describe a unique case of a child diagnosed with extrahepatic biliary fibrotic stenosis likely secondary to a prior spontaneous biliary tract perforation (SBTP), presenting with an unusual extremely severe LpX-mediated hypercholesterolemia. Cholesterol levels of this patient were 2 times higher than the levels previously reported in the biliary atresia literature, akin only to those of few other cases of Alagille syndrome (8).

## Case report

Our patient is a developmentally normal 18-month boy, born to unrelated healthy parents as a full-term infant with no complications during gestation or delivery. He was admitted for recent jaundice, pale stools, hyperchromic urines, and pruritus. He was previously healthy and was not taking medications. Clinical examination showed moderate hepatomegaly, scratching signs on the skin, and jaundice. His height was at the 10th percentile (77 cm) for age and sex, weight at the >5th percentile (8.440 kg), weight/height ratio at the <5th percentile, and blood pressure at the 50th percentile (85/45 mmHg). As shown in Table 1, laboratory tests at entry revealed marked conjugated hyperbilirubinemia and hypercholanemia (total bile acids 100 folds upper normal values) along with increased serum hepatobiliary enzymes.

**Table 1:** Cholestasis parameters and lipid profile before/after surgery, and during follow up.

	**Normal values**	**Before surgery**	**10 days after surgery**	**6 months** **after surgery**	**12 months after surgery**
Total cholesterol (mg/dL)	< 200	1730	169	153	268
Unesterified cholesterol (mg/dL)	< 60	1456	46	39	101
Unesterified cholesterol to total cholesterol ratio	< 0.30	0.84	0.27	0.25	0.38
Triglycerides (mg/dL)	< 150	176	130	115	285
AST (U/L)	<40	173	84	55	75
ALT (U/L)	<41	165	65	58	85
GGT (U/L)	<60	300	192	179	200
TB (mg/dL)	<1	9.70	0.35	0.5	0.8
DB (mg/dL)	0–0.4	5.30	0.15	0.25	0.2
Bile Acids (μmol/L)	<6.0	643.8	63.3	15	62
HDL-cholesterol (mg/dL)	≥60	6	56	93	84
Non HDL-cholesterol (mg/dL)	<100	1724	113	60	184
Apolipoprotein B (mg/dL)	70–150	165	79	82	121
Phospholipids (mg/dL)	<200	3073	259	135	385
Lipoprotein X	Absent	Present	Absent	Absent	Absent

ALT, alanine aminotransferase; AST, aspartate aminotransferase; DB, Direct bilirubin; GGT, gamma glutamyl transpeptidase; HDL, High density lipoprotein; TB, total bilirubin.

**Figure 1:**
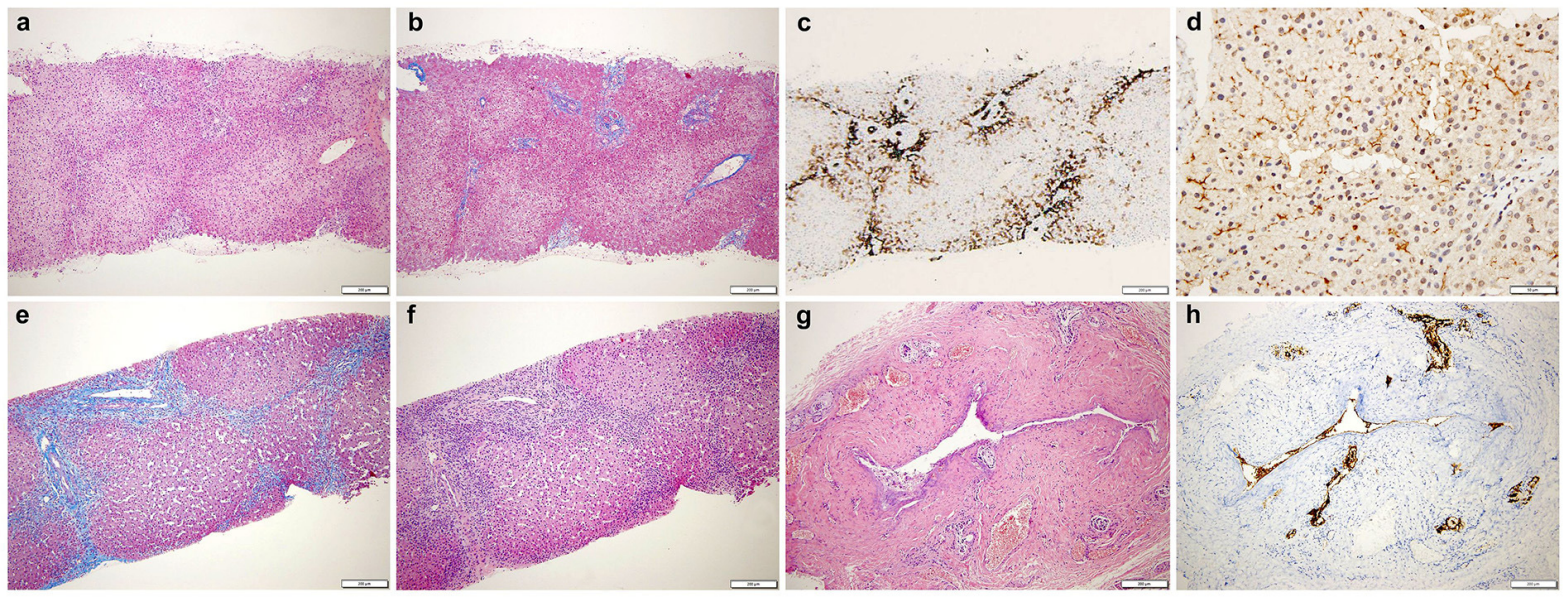
(Top) Liver biopsy shows: **(a)** preserved architecture with mild portal inflammation (HE, 4x). **(b)** fibroedematous portal tracts (Masson trichrome, 4x). **(c)** CK7 immunostaining displays interlobular biliary ducts and diffuse abnormal staining of periportal hepatocytes (CK7, 4x). **(d)** MDR3 is normally expressed at the canalicular pole of the hepatocytes (MDR3, 20x). (Bottom) **(e)** Increased inflammation (HE, 4x) and **(f)** fibrosis with fibrous septae (Masson trichrome, 4x). **(g)** The extrahepatic biliary duct shows a thick fibrotic wall. Small siero-mucous glands are present within the fibrous wall. No neoplastic cells are evident (HE, 10x). **(h)** CK7 shows residual epithelial of the main lumen (CK7, 10x). HE, hematoxylin and eosin; CK7, cytokeratin 7; MDR3, multidrug resistance protein 3.

Search for hepatotropic infectious, endocrine, metabolic, and toxic causes of severely hyperlipidemic cholestasis included an extensive gene panel (Cholestasis, Version 1.110 - Genomics England PanelApp) (9) (Supplementary Table S1) which resulted negative. As an initial ultrasonographic study was normal except for mild hepatomegaly, a percutaneous needle liver biopsy was performed with two main clinical hypotheses: a bile ducts paucity or a flippase deficiency, two cholestatic conditions with elevated GGT. The biopsy showed portal tracts with interlobular biliary ducts with features of obstructive cholangitis with biliary fibrosis and bile ducts proliferation (Figures 1a–c). An immunohistochemical staining with anti- Multidrug Resistance protein 3 (MDR3 Antibody, clone P3 II-26 | MAB4140—EMD Millipore, 1:100) was performed and the label was normally expressed at the canalicular pole of the hepatocytes (Figure 1d), ruling out a flippase deficiency.

**Figure 2:**
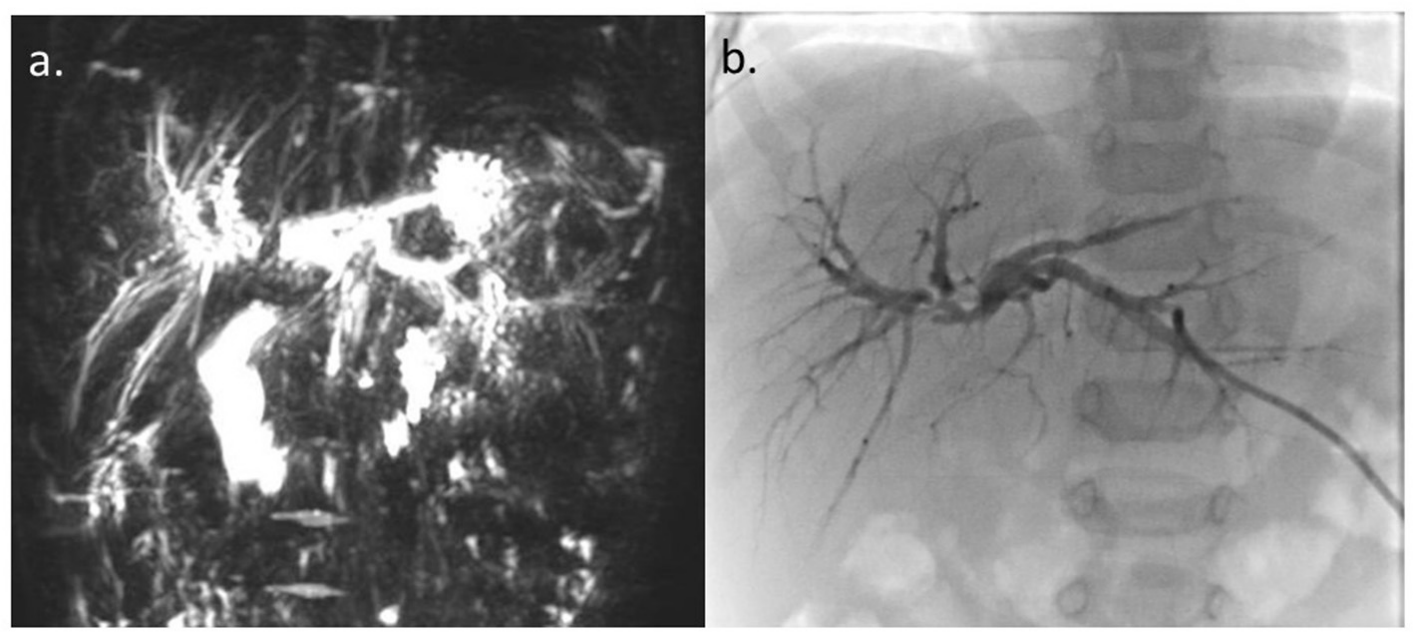
**(a)** Magnetic resonance cholangiopancreatography. **(b)** Percutaneous transhepatic cholangiography. Dilation of intrahepatic ducts and of the two hepatic ducts with regular profiles, abruptly stopping at the confluence level, without appreciation of the choledochus and the gallbladder.

Further imaging studies [magnetic resonance cholangiopancreatography] showed a dilation of the intrahepatic ducts and of the two hepatic ducts, abruptly stopping at the confluence level (Figure 2a).

**Figure 3:**
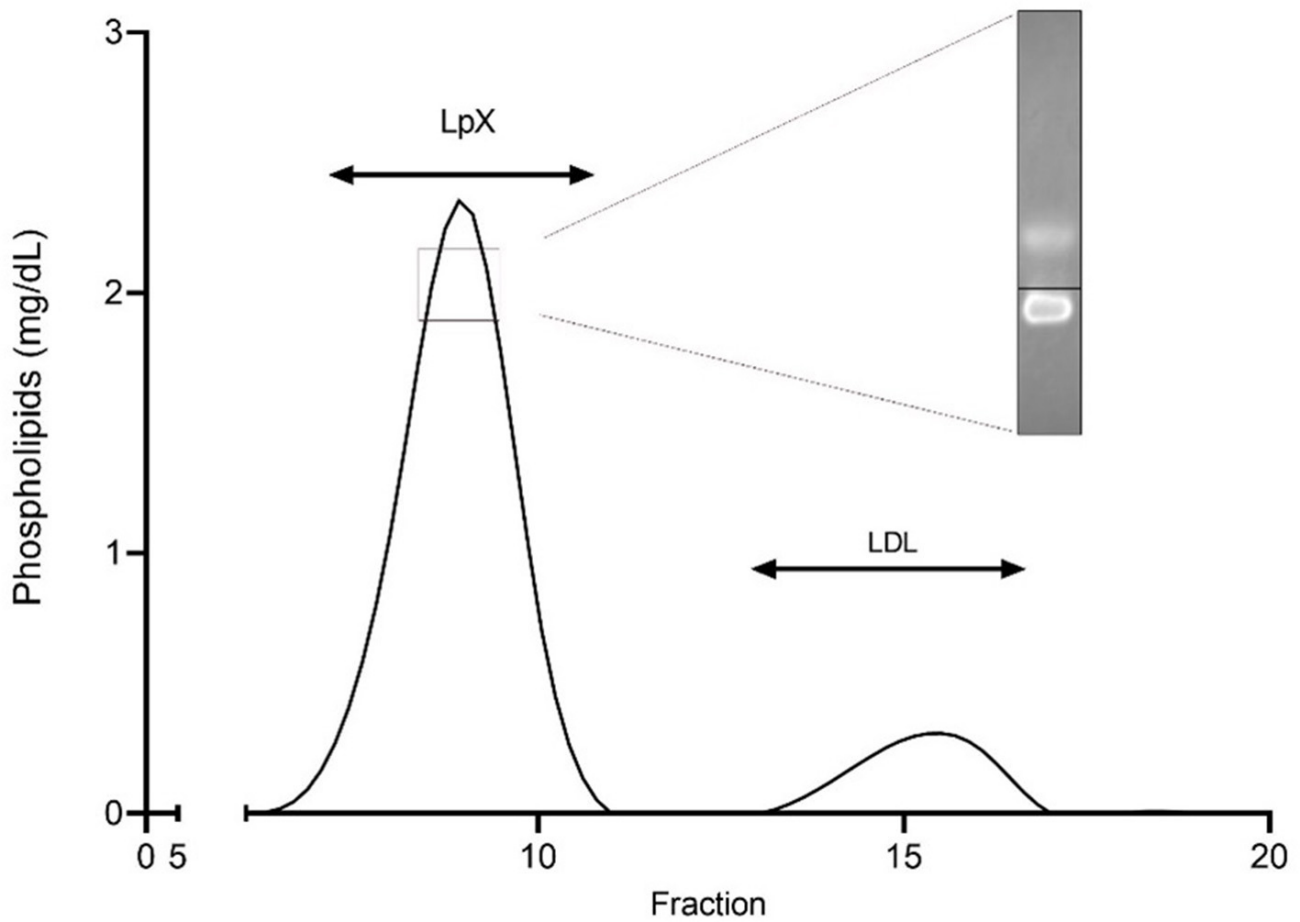
Separation of the 1.020–1.063 g/ml plasma fraction by fast protein liquid chromatography (FPLC). The 1.020–1.063 g/ml density fraction, corresponding to lipoprotein X and LDL, was separated by FPLC and analyzed for phospholipid content. Insert shows cathodic migration of Lp X stained with filipin at agarose gel electrophoresis. Black line indicates origin.

The concomitant finding of a particularly worrying, extremely marked, hyperlipidemia (total cholesterol 1730 mg/dl, triglycerides 176 mg/dl) resistant to choleretic therapy with ursodeoxycholic acid (up to 25 mg/kg/day) prompted the exam of our patient thyroid hormones and his parents' lipids serum levels, which returned normal and thus excluded the diagnosis of an associated hypothyroidism or homozygous familial hypercholesterolemia, respectively. A nephrotic syndrome was excluded as well. A comprehensive study of patient's blood lipids was therefore undertaken. In brief, fasting blood samples were collected and plasma was separated by low-speed centrifugation at 4°C. Elevated plasma unesterified to total cholesterol ratio and phospholipids, together with disproportionally low serum apolipoprotein B levels, supported the likely presence of the abnormal LpX. Since LpX's density and size are similar to LDL and VLDL, respectively, to confirm its presence in plasma we first separated by sequential ultracentrifugation the 1.020–1.063 g/mL lipoprotein fraction, which was subsequently analyzed by Fast Protein Liquid Chromatography, as previously described (10) (Figure 3). Presence of LpX was additionally confirmed by electrophoresis using Sebia agarose gels, followed by Filipin staining of unesterified cholesterol. Fluorescent spot corresponding to LpX on the gel was monitored by ChemiDoc (BioRad,Hercules,CA,USA) (11).

Percutaneous transhepatic cholangiography (PTC) confirmed stenosis of the confluence of the right and left hepatic ducts and failure to visualize the extrahepatic biliary tree below the stenosis (Figure 2b). An external biliary stent placed across the confluence through the stenotic tract, was not able to pass into the main bile duct. Intrahepatic biliary tree looked normal, except for mild-to-moderate dilatation. Surgical exploration revealed the presence of a focal stenosis of the common hepatic duct and of the confluence of the right and left bile ducts, without clear signs of a previous SBTP. The gallbladder and the common bile duct looked normal. The extrahepatic biliary tract was resected up to the carrefour, where the external biliary stent was present. The extemporaneous histology excluded the presence of neoplastic cells and intraoperative cholangiography confirmed the PTC findings. Biliary continuity was restored by anastomosing the right and left bile ducts and the posterior wall of the carrefour to a defunctionalized ileal loop according to Roux. The postoperative course was without complications, and a transhepatic biliary stent cholangiography performed 4 weeks after surgery documented a normal morphology of the intrahepatic biliary tract and the regular transit of the contrast medium through the biliodigestive anatomosis.

At the definitive histologic exam, the liver was characterized by cholangitis and biliary fibrosis (Figures 1e,f). The removed extrahepatic biliary tree displayed a fibrotic thickened wall with only a slim lumen (Figures 1g,h); the gallbladder, normally shaped, showed chronic cholecystitis. As shown in Table 1, surgery was followed by the rapid disappearance of LpX, along with stable normalization of serum lipids profile, bilirubin and bile acids, and a progressive reduction of hepatobiliary enzymes.

## Discussion

In adulthood, LpX may be observed in plasma of patients with cholestatic hepatitis, primary biliary cholangitis, primary sclerosing cholangitis, (12) graft vs. host disease of the liver (13) or after lipid infusion (e.g., Intralipid) (14) and tend to resolve after removal of the causative factor/s. In pediatrics, high levels of LpX have been emphasized especially in the setting of the severe hyperlipidemia observed in patients with intrahepatic cholestasis due to syndromic paucity of intralobular bile ducts (Alagille syndrome), and in children with biliary atresia where LpX related hypercholesterolemia appears higher than in all intrahepatic cholestasis except Alagille syndrome (5). Our report draws attention to the possibility that extremely high cholesterol levels in a cholestatic child may represent LpX-related hypercholesterolemia also in conditions other than Alagille syndrome.

Regarding intrahepatic causes of cholestasis, our patient's clinical features, genetic study and liver histology were not consistent with either a syndromic or a not syndromic paucity of biliary ducts but showed instead a picture orienting toward a possible high GGT cholestasis due to the MDR3 defect. This however was not confirmed by the immune-histochemical and liver panel study. As for extrahepatic causes of cholestasis, while the diagnosis of a typical biliary atresia was unlikely due to our patient's age, the imaging study and anatomical finding at surgery could still play in favor of the sequel of a possible prior SBTP. This is a quite uncommon condition with a still unclear etiology probably due to multiple concomitant causes including developmental intrinsic weakness of the duct wall/pancreatic biliary malunion/distal bile duct stenoses/ ischemic insult (15). Although our patient had an age corresponding to that of the majority of SBTP cases reported in the literature to date (14), his clinical presentation differs mainly due to the lack of evidence of previous manifest abdominal distension/ascites. On the other hand, his age at presentation was much higher than those of the 8- and 3-month infants with late presentation-acquired biliary atresia reported by Davenport et al. (16) and Koshinaga et al. (17), respectively. In both cases, these authors suggested that the acquired obliterative cholangiopathy of their patients could have been a likely result of the chronological progression of the sclerosing effect of a bile leak from an unnoticed spontaneous bile duct perforation.

Circulating LpX with cholesterol accumulation and impaired lipoprotein homeostasis in cholestatic diseases derives from several concurrent factors, i.e., spillover of biliary lipids into plasma, accrual of phospholipids and unesterified cholesterol in serum, bile acid suppressed lecithin: cholesterol acyltransferase (LCAT) activity, and disturbed enterohepatic circulation of bile acids which reduces farnesoid X receptor-mediated feedback (4, 18). After integrating also small quantities of triglycerides, apo-C and esterified cholesterol it attaches with a non-covalent binding to the surface of a protein core of albumin and becomes a “mature” LpX. Although this distinctive lipoprotein has similar density to LDL, it is lacking apolipoprotein B and its metabolism is different from that of LDL because cannot be cleared by LDL receptors. As a result, LpX cannot trigger the negative feedback in cholesterol production (19, 20) and its removal from plasma relays only in the reticuloendothelial system (mainly spleen). Diagnostic clues for the presence of LpX are provided by normal/low apolipoprotein B level (20) which should prompt the measurement of unesterified cholesterol levels and unesterified to total cholesterol ratio calculation. In our patient LpX was suspected by the presence of significantly high unesterified cholesterol and even more strongly by increased unesterified to total cholesterol ratio. The presence of LpX was confirmed by the analysis of the 1.020–1.063 g/ml density fraction, normally corresponding to LDL and eventually also containing LpX. The separation of this fraction by FPLC clearly demonstrated the presence of LpX which largely explains the hypercholesterolemia.

LpX-mediated hypercholesterolemia is clinically problematic as it does not respond to usual lipid lowering therapies regulating LDL-cholesterol levels by binding to liver LDL receptors and stimulating their intracellular degradation. Moreover, in case of drugs with prevalent hepatic/biliary elimination (e.g., most statins) cholestasis may even favor their toxic concentrations (21, 22). Still, intimal-medial thickness and arterial wall stiffness studies conducted in patients with Alagille syndrome suggest that LpX may have scarce atherogenic effects (7) likely due to its property of preventing LDL oxidation (23) or to the molecule's larger size not allowing to cross the arterial endothelium (24). Although this may raise the question if treatment for LpX-mediated hypercholesterolemia warrants medical therapeutic attempts its correct recognition still requires clinicians' and laboratories' attention due to the marked risk of disfiguring skin xanthomas, nephrotoxicity (25), and pulmonary embolism as a result of blood hyperviscosity. This may require periodic LDL apheresis or plasma exchange which have been reported effective or even liver transplantation (25–27). Moreover, spurious laboratory abnormalities of serum electrolytes and total proteins (e.g., pseudo-hyponatremia and artifactual hyperproteinemia) described in patients with LpX should be equally noticed to avoid improper treatments (27).

In conclusion, we report a unique case of an 18-month patient diagnosed with abrupt onset of obstructive cholestatic jaundice due to biliary stenosis resembling those secondary to a possible prior spontaneous perforation of biliary tract. His extremely high levels of LpX-mediated hypercholesterolemia were more than two times higher than those previously described in other cases of pediatric extrahepatic cholestasis (namely in BA) and were akin only to those reported in few patients with Alagille syndrome. Its correct recognition is important to differentiate LpX-mediated hypercholesterolemia from elevated LDL concentrations as this may impact the therapeutic management.

## Data availability statement

The original contributions presented in the study are included in the article/Supplementary material, further inquiries can be directed to the corresponding author.

## Ethics statement

Ethical review and approval was not required for the study on human participants in accordance with the local legislation and institutional requirements. Written informed consent to participate in this study was provided by the participants' legal guardian/next of kin.

## Author contributions

RC, PV, AP, and CM contributed to the conception and design of the work. PV and CM wrote the draft. CP, MT, and LC carried out lipids studies. PF carried out histological studies. MS performed the surgical intervention. PV supervised the entire project. RC, CP, AP, MT, PF, MS, PV, LC, and CM discussed the results and contributed to the final manuscript. All authors read and approved the manuscript.

## Conflict of interest

The authors declare that the research was conducted in the absence of any commercial or financial relationships that could be construed as a potential conflict of interest.

## Publisher's note

All claims expressed in this article are solely those of the authors and do not necessarily represent those of their affiliated organizations, or those of the publisher, the editors and the reviewers. Any product that may be evaluated in this article, or claim that may be made by its manufacturer, is not guaranteed or endorsed by the publisher.
