# Supplementary material for: Case report: Unusual and extremely severe lipoprotein X-mediated hypercholesterolemia in extrahepatic pediatric cholestasis
Source: Front Pediatr. 2022 Aug 4;10:969081. doi: 10.3389/fped.2022.969081 (PMC9386286; doi:10.3389/fped.2022.969081)
Supplement: Supplementary file 1 [file Data_Sheet_1.PDF]

The latest signed off version for the GMS is v1.21. The current version, shown here, may differ from the signed-off version.

Cholestasis (Version 1.110)

Relevant disorders: R171
Panel types: GMS Rare Disease Virtual, GMS Rare Disease, GMS signed-off
Latest signed off version: v1.21 (20 Aug 2020)

78 Entities

78 reviewed, 61 green

List	Entity	Reviews	Mode of inheritance	Details
**Filter Entities**				
78 Entities				
GreenGreen List (high evidence)	**ABCB11**	3 reviews 3 green	BIALLELIC, autosomal or pseudoautosomal	**Sources**<br>• Expert Review Green<br>• NHS GMS<br>• Other<br>**Phenotypes**<br>• Familial Intrahepatic Cholestasis<br>• Cholestasis, progressive familial intrahepatic 2, 601847<br>• Cholestasis, Progressive Familial Intrahepatic 2<br>• PFIC2<br>• Cholestasis, benign recurrent intrahepatic, 2, 605479

List	Entity	Reviews	Mode of inheritance	Details
**Filter Entities**				
78 Entities				
				• Neonatal and Adult Cholestasis<br>**Tags**
GreenGreen List (high evidence)	**ABCB4**	4 reviews 3 green	BOTH monoallelic and biallelic, autosomal or pseudoautosomal	**Sources**<br>• Expert Review Green<br>• NHS GMS<br>• Other<br>**Phenotypes**<br>• Progressive Familial Intrahepatic Cholestasis<br>• modifier in other forms of genetic cholestasis<br>• Familial Intrahepatic Cholestasis<br>• gallstones<br>• cholelithiasis<br>• PFIC<br>• PFIC3<br>• Cholestasis, progressive familial intrahepatic 3, 602347<br>• Cholestasis, intrahepatic, of pregnancy, 3, 614972<br>• Neonatal and Adult Cholestasis<br>• Cholestasis, Progressive Familial Intrahepatic 3<br>**Tags**
GreenGreen List (high evidence)	**ABCC2**	4 reviews	BIALLELIC, autosomal or pseudoautosomal	**Sources**<br>• Emory Genetics Laboratory

List	Entity	Reviews	Mode of inheritance	Details
**Filter Entities**				
78 Entities				
		2 green		• Expert Review Green<br>• NHS GMS<br>• Other<br>• Victorian Clinical Genetics Services<br>**Phenotypes**<br>• modifier in biliary atresia<br>• Dubin Johnson syndrome<br>• Cholestasis<br>• intrahepatic cholestasis of pregnancy<br>• Dubin-Johnson syndrome, 237500<br>**Tags**
GreenGreen List (high evidence)	ADK	2 reviews 1 green	BIALLELIC, autosomal or pseudoautosomal	**Sources**<br>• Expert list<br>• Expert Review Green<br>**Phenotypes**<br>• Hypermethioninemia due to adenosine kinase deficiency, OMIM:614300, MONDO:0013676<br>**Tags**
GreenGreen List (high evidence)	AKR1D1	4 reviews 2 green	BIALLELIC, autosomal or pseudoautosomal	**Sources**<br>• Emory Genetics Laboratory<br>• Expert Review Green<br>• NHS GMS<br>• Other

List	Entity	Reviews	Mode of inheritance	Details

Filter Entities

78 Entities

				• UKGTN<br>• Victorian Clinical Genetics Services<br><br>**Phenotypes**<br><br>• Bile acid synthesis defect, congenital, 2 235555<br>• fat soluble vitamin deficiency<br>• liver failure<br>• bile salt synthesis defect<br>• Bile acid synthesis defect, congenital, 2<br>• Neonatal and Adult Cholestasis<br>• cholestasis<br><br>**Tags**
GreenGreen List (high evidence)	ALDOB	4 reviews<br>1 green	BIALLELIC, autosomal or pseudoautosomal	**Sources**<br><br>• Emory Genetics Laboratory<br>• Expert Review Green<br>• NHS GMS<br>• Other<br>• UKGTN<br>• Victorian Clinical Genetics Services<br><br>**Phenotypes**<br><br>• acute liver failure<br>• Neonatal and Adult Cholestasis<br>• Fructose intolerance, hereditary<br><br>**Tags**

List	Entity	Reviews	Mode of inheritance	Details
**Filter Entities**				
78 Entities				
GreenGreen List (high evidence)	AMACR	4 reviews 1 green	BIALLELIC, autosomal or pseudoautosomal	**Sources**<br>• Expert Review Green<br>• NHS GMS<br>• Other<br>**Phenotypes**<br>• Neonatal and Adult Cholestasis<br>• Bile acid synthesis defect, congenital, 4 214950<br>**Tags**
GreenGreen List (high evidence)	ATP7B	2 reviews 1 green	BIALLELIC, autosomal or pseudoautosomal	**Sources**<br>• Expert list<br>• Expert Review Green<br>**Phenotypes**<br>• Wilson disease, 277900<br>**Tags**
GreenGreen List (high evidence)	ATP8B1	4 reviews 3 green	BOTH monoallelic and biallelic, autosomal or pseudoautosomal	**Sources**<br>• Expert Review Green<br>• NHS GMS<br>• Other<br>**Phenotypes**<br>• Familial Intrahepatic Cholestasis<br>• Cholestasis, intrahepatic, of pregnancy, 1, 147480

List	Entity	Reviews	Mode of inheritance	Details
**Filter Entities** 78 Entities				
				• Cholestasis, Progressive Familial Intrahepatic 1<br>• Cholestasis, benign recurrent intrahepatic, 243300<br>• Cholestasis, progressive familial intrahepatic 1, 211600<br>• Neonatal and Adult Cholestasis<br>**Tags**
GreenGreen List (high evidence)	BAAT	3 reviews 2 green	BIALLELIC, autosomal or pseudoautosomal	**Sources**<br>• Expert Review Green<br>• NHS GMS<br>• Other<br>**Phenotypes**<br>• Hypercholanemia, Familial<br>• fat soluble vitamin deficiency<br>• Hypercholanemia, familial, 607748<br>• cholestasis<br>• Neonatal and Adult Cholestasis<br>**Tags**
GreenGreen List (high evidence)	BCS1L	4 reviews 2 green	BIALLELIC, autosomal or pseudoautosomal	**Sources**<br>• Expert list<br>• Expert Review Green<br>• NHS GMS<br>• Other<br>**Phenotypes**

List	Entity	Reviews	Mode of inheritance	Details
**Filter Entities** 78 Entities				
				• Cholestasis<br>• GRACILE syndrome<br><br>**Tags**
GreenGreen List (high evidence)	CFTR	2 reviews<br>1 green	BIALLELIC, autosomal or pseudoautosomal	**Sources**<br>• Expert list<br>• Expert Review Green<br><br>**Phenotypes**<br>• Cholestasis<br>• Neonatal and Adult Cholestasis<br>• Cystic fibrosis, OMIM:219700, MONDO:0009061<br>• {Pancreatitis, hereditary}, OMIM:167800<br><br>**Tags**
GreenGreen List (high evidence)	CLDN1	3 reviews<br>2 green	BIALLELIC, autosomal or pseudoautosomal	**Sources**<br>• Expert Review Green<br>• NHS GMS<br>• Other<br><br>**Phenotypes**<br>• ichthyosis-hypotrichosis-sclerosing cholangitis<br>• Ichthyosis, leukocyte vacuoles, alopecia and sclerosing cholangitis, 607626<br>• Neonatal and Adult Cholestasis

List	Entity	Reviews	Mode of inheritance	Details
**Filter Entities**				
78 Entities				
				• NISCH syndrome<br>• Neonatal ichthyosis sclerosing cholangitis (NISCH) syndrome<br><br>**Tags**
GreenGreen List (high evidence)	COG7	2 reviews<br>1 green	BIALLELIC, autosomal or pseudoautosomal	**Sources**<br>• Expert list<br>• Expert Review Green<br><br>**Phenotypes**<br>• Congenital disorder of glycosylation, type IIe , 608779<br><br>**Tags**
GreenGreen List (high evidence)	CYP27A1	4 reviews<br>2 green	BIALLELIC, autosomal or pseudoautosomal	**Sources**<br>• Emory Genetics Laboratory<br>• Expert Review Green<br>• NHS GMS<br>• Other<br>• UKGTN<br>• Victorian Clinical Genetics Services<br><br>**Phenotypes**<br>• Severe neonatal cholestasis<br>• Cerebrotendinous xanthomatosis, 213700<br><br>**Tags**

List	Entity	Reviews	Mode of inheritance	Details

Filter Entities

78 Entities

List	Entity	Reviews	Mode of inheritance	Details
GreenGreen List (high evidence)	CYP7A1	5 reviews 1 green 2 red	BIALLELIC, autosomal or pseudoautosomal	**Sources**<br>• Expert Review Green<br>• NHS GMS<br>• Other<br>**Phenotypes**<br>• Bile acid synthesis defect, congenital, 3<br>• Neonatal and Adult Cholestasis<br>**Tags**<br>• for-review<br>• gene-checked
GreenGreen List (high evidence)	CYP7B1	5 reviews 1 green	BIALLELIC, autosomal or pseudoautosomal	**Sources**<br>• Emory Genetics Laboratory<br>• Expert Review Green<br>• NHS GMS<br>• Other<br>• UKGTN<br>• Victorian Clinical Genetics Services<br>**Phenotypes**<br>• Bile acid synthesis defect, congenital, 3, 613812<br>• Neonatal and Adult Cholestasis

List	Entity	Reviews	Mode of inheritance	Details
**Filter Entities**				
78 Entities				
				**Tags**
GreenGreen List (high evidence)	DCDC2	4 reviews 2 green	BIALLELIC, autosomal or pseudoautosomal	**Sources**<br>• Emory Genetics Laboratory<br>• Expert Review Green<br>• NHS GMS<br>• Other<br>• Victorian Clinical Genetics Services<br>**Phenotypes**<br>• Sclerosing cholangitis, neonatal, 617394<br>• PFIC type 5<br>• Neonatal sclerosis cholangitis<br>• Neonatal and Adult Cholestasis<br>**Tags**
GreenGreen List (high evidence)	DGUOK	2 reviews 1 green	BIALLELIC, autosomal or pseudoautosomal	**Sources**<br>• Expert list<br>• Expert Review Green<br>**Phenotypes**<br>• Mitochondrial DNA depletion syndrome 3 (hepatocerebral type), 251880<br>**Tags**
GreenGreen List (high evidence)	FAH	3 reviews	BIALLELIC, autosomal or pseudoautosomal	**Sources**<br>• Expert Review Green

List	Entity	Reviews	Mode of inheritance	Details
**Filter Entities** 78 Entities				
		1 green		• NHS GMS<br>**Phenotypes**<br>• Neonatal and Adult Cholestasis<br>• Tyrosinaemia, Type 1, 276700<br>• Cholestasis<br>**Tags**
GreenGreen List (high evidence)	GALE	2 reviews 1 green	BIALLELIC, autosomal or pseudoautosomal	**Sources**<br>• Expert Review<br>• Expert Review Green<br>**Phenotypes**<br>• Galactose epimerase deficiency, OMIM:230350<br>• MONDO:0009257<br>**Tags**
GreenGreen List (high evidence)	GALK1	2 reviews	BIALLELIC, autosomal or pseudoautosomal	**Sources**<br>• Expert Review<br>• Expert Review Green<br>**Phenotypes**<br>• Galactokinase deficiency with cataracts, OMIM:230200<br>• MONDO:0009255 Edit<br>**Tags**

List	Entity	Reviews	Mode of inheritance	Details
**Filter Entities** 78 Entities				
				• Q1_22_NHS_review<br>• Q2_22_expert_review<br>• Q2_22_rating
GreenGreen List (high evidence)	GALM	2 reviews 1 green	BIALLELIC, autosomal or pseudoautosomal	**Sources**<br>• Expert Review Green<br>• Literature<br>**Phenotypes**<br>• Galactosemia IV, OMIM:618881<br>• MONDO:0030105<br>**Tags**
GreenGreen List (high evidence)	GALT	2 reviews 1 green	BIALLELIC, autosomal or pseudoautosomal	**Sources**<br>• Expert list<br>• Expert Review Green<br>**Phenotypes**<br>• Galactosemia, OMIM:230400<br>• MONDO:0018116<br>**Tags**

List	Entity	Reviews	Mode of inheritance	Details
**Filter Entities** 78 Entities				
GreenGreen List (high evidence)	GBA	3 reviews 1 green	BIALLELIC, autosomal or pseudoautosomal	**Sources**<br>• Expert list<br>• Expert Review Green<br>**Phenotypes**<br>• Gaucher disease, perinatal lethal 608013<br>• Gaucher disease, type I 230800<br>• Gaucher disease, type II 230900<br>• Gaucher disease, type III 231000<br>• Gaucher disease, type IIIC 231005<br>**Tags**<br>• new-gene-name
GreenGreen List (high evidence)	GBE1	2 reviews	BIALLELIC, autosomal or pseudoautosomal	**Sources**<br>• Expert list<br>• Expert Review Green<br>**Phenotypes**<br>• Glycogen storage disease IV, OMIM:232500<br>**Tags**<br>• Q1_22_NHS_review

List	Entity	Reviews	Mode of inheritance	Details
**Filter Entities** 78 Entities				
				• Q2_22_expert_review<br>• Q2_22_rating
GreenGreen List (high evidence)	HADHA	2 reviews 1 green	BIALLELIC, autosomal or pseudoautosomal	**Sources**<br>• Expert list<br>• Expert Review Green<br>**Phenotypes**<br>• LCHAD deficiency, OMIM:609016, MONDO:0012173<br>**Tags**
GreenGreen List (high evidence)	HNF1B	2 reviews 1 green	MONOALLELIC, autosomal or pseudoautosomal, NOT imprinted	**Sources**<br>• Expert list<br>• Expert Review Green<br>**Phenotypes**<br>• Renal cysts and diabetes syndrome, 137920<br>**Tags**
GreenGreen List (high evidence)	HSD3B7	3 reviews 2 green	BIALLELIC, autosomal or pseudoautosomal	**Sources**<br>• Expert Review Green<br>• NHS GMS<br>• Other

List	Entity	Reviews	Mode of inheritance	Details
**Filter Entities**				
78 Entities				
				**Phenotypes**<br>• Bile acid sythesis defect, congenital, 1 607765<br>• Neonatal and Adult Cholestasis<br>**Tags**
GreenGreen List (high evidence)	JAG1	6 reviews<br>4 green	MONOALLELIC, autosomal or pseudoautosomal, NOT imprinted	**Sources**<br>• Expert Review Green<br>• NHS GMS<br>• Other<br>**Phenotypes**<br>• Alagille syndrome 1, OMIM:118450<br>• Neonatal and Adult Cholestasis<br>**Tags**
GreenGreen List (high evidence)	KIF12	2 reviews<br>1 green	BIALLELIC, autosomal or pseudoautosomal	**Sources**<br>• Expert Review Green<br>• Literature<br>**Phenotypes**<br>• Cholestasis<br>• High Gamma-Glutamyltransferase (GGT)<br>**Tags**

List	Entity	Reviews	Mode of inheritance	Details
**Filter Entities**				
78 Entities				
GreenGreen List (high evidence)	LIPA	2 reviews 1 green	BIALLELIC, autosomal or pseudoautosomal	**Sources**<br>• Expert list<br>• Expert Review Green<br>**Phenotypes**<br>• lysosomal acid lipase deficiency<br>• Wolman disease, OMIM:278000, MONDO:0019148<br>• Cholesteryl ester storage disease, OMIM:278000, MONDO:0019149<br>• Neonatal and Adult Cholestasis<br>• cholestasis<br>**Tags**
GreenGreen List (high evidence)	MPI	2 reviews 1 green	BIALLELIC, autosomal or pseudoautosomal	**Sources**<br>• Expert list<br>• Expert Review Green<br>**Phenotypes**<br>• Congenital disorder of glycosylation, type Ib, OMIM:602579<br>• MPI-CDG, MONDO:0011257<br>**Tags**
GreenGreen List (high evidence)	MPV17	2 reviews 1 green	BIALLELIC, autosomal or pseudoautosomal	**Sources**<br>• Expert list<br>• Expert Review Green

List	Entity	Reviews	Mode of inheritance	Details
**Filter Entities** 78 Entities				
				**Phenotypes**<br>• Mitochondrial DNA depletion syndrome 6 (hepatocerebral type), 256810<br>**Tags**
GreenGreen List (high evidence)	MVK	2 reviews 1 green	BIALLELIC, autosomal or pseudoautosomal	**Sources**<br>• Expert list<br>• Expert Review Green<br>**Phenotypes**<br>• Mevalonic aciduria, OMIM:610377<br>**Tags**
GreenGreen List (high evidence)	MYO5B	4 reviews 2 green 1 red	BIALLELIC, autosomal or pseudoautosomal	**Sources**<br>• Expert list<br>• Expert Review Green<br>• NHS GMS<br>**Phenotypes**<br>• Diarrhea 2, with microvillus atrophy, OMIM:251850<br>**Tags**
GreenGreen List (high evidence)	NBAS	3 reviews 1 green 1 red	BIALLELIC, autosomal or pseudoautosomal	**Sources**<br>• Expert Review Green<br>• Literature<br>**Phenotypes**

List	Entity	Reviews	Mode of inheritance	Details
Filter Entities<br>78 Entities				
				• Infantile liver failure syndrome 2, OMIM:616483<br>**Tags**
GreenGreen List (high evidence)	NOTCH2	5 reviews<br>3 green	MONOALLELIC, autosomal or pseudoautosomal, NOT imprinted	**Sources**<br>• Expert Review Green<br>• NHS GMS<br>• Other<br>**Phenotypes**<br>• Alagille syndrome 2<br>• Neonatal and Adult Cholestasis<br>**Tags**
GreenGreen List (high evidence)	NPC1	4 reviews<br>3 green	BIALLELIC, autosomal or pseudoautosomal	**Sources**<br>• Expert Review Green<br>• NHS GMS<br>• Other<br>**Phenotypes**<br>• Niemann-Pick disease, type D, 257220<br>• Niemann-Pick disease type C1, 257220<br>• Neonatal and Adult Cholestasis<br>**Tags**
GreenGreen List (high evidence)	NPC2	4 reviews	BIALLELIC, autosomal or pseudoautosomal	**Sources**<br>• Expert Review Green<br>• NHS GMS

List	Entity	Reviews	Mode of inheritance	Details

Filter Entities

78 Entities

List	Entity	Reviews	Mode of inheritance	Details
		3 green		• Other<br>**Phenotypes**<br>• Neonatal and Adult Cholestasis<br>• Niemann-Pick disease type C2, 607625<br>**Tags**
GreenGreen List (high evidence)	NR1H4	4 reviews 3 green	BIALLELIC, autosomal or pseudoautosomal	**Sources**<br>• Expert Review Green<br>• NHS GMS<br>• Other<br>**Phenotypes**<br>• ciliopathy<br>• Cholestasis, Progressive Familial Intrahepatic 5<br>• modifier of other genetic cholestatic conditions<br>• Neonatal and Adult Cholestasis<br>• Cholestasis, progressive familial intrahepatic 5, 617049<br>**Tags**
GreenGreen List (high evidence)	PEX1	5 reviews 1 green 1 red	BIALLELIC, autosomal or pseudoautosomal	**Sources**<br>• Expert Review Green<br>• NHS GMS<br>• Other<br>**Phenotypes**

List	Entity	Reviews	Mode of inheritance	Details
**Filter Entities** 78 Entities				
				• Peroxisome Biogenesis Disorder 1A (Zellweger), 214100<br>• Zellweger syndrome<br>• Neonatal and Adult Cholestasis<br><br>**Tags**
GreenGreen List (high evidence)	PEX12	3 reviews 1 green 1 red	BIALLELIC, autosomal or pseudoautosomal	**Sources**<br>• Expert Review Green<br>• NHS GMS<br>• Other<br><br>**Phenotypes**<br>• Peroxisome biogenesis disorder 3B 266510<br>• Peroxisome biogenesis disorder 3A (Zellweger) 614859<br><br>**Tags**
GreenGreen List (high evidence)	PEX26	2 reviews 1 green	BIALLELIC, autosomal or pseudoautosomal	**Sources**<br>• Expert Review Green<br>• NHS GMS<br>• Other<br><br>**Phenotypes**<br>• Peroxisome biogenesis disorder 7A (Zellweger)614872<br><br>**Tags**

List	Entity	Reviews	Mode of inheritance	Details
**Filter Entities**				
78 Entities				
GreenGreen List (high evidence)	PEX6	3 reviews 1 green 1 red	BIALLELIC, autosomal or pseudoautosomal	**Sources**<br>• Expert Review Green<br>• NHS GMS<br>• Other<br>**Phenotypes**<br>• Peroxisome biogenesis disorder 4A (Zellweger) 614862<br>**Tags**
GreenGreen List (high evidence)	PKHD1	2 reviews 1 green	BIALLELIC, autosomal or pseudoautosomal	**Sources**<br>• Expert list<br>• Expert Review Green<br>**Phenotypes**<br>• Polycystic kidney disease 4, with or without hepatic disease, OMIM:263200<br>• MONDO:0044327<br>**Tags**
GreenGreen List (high evidence)	POLG	2 reviews 1 green	BIALLELIC, autosomal or pseudoautosomal	**Sources**<br>• Expert list<br>• Expert Review Green<br>**Phenotypes**<br>• Mitochondrial DNA depletion syndrome 4A (Alpers type),

List	Entity	Reviews	Mode of inheritance	Details
**Filter Entities** 78 Entities				
				OMIM:203700, MONDO:0008758<br>**Tags**
GreenGreen List (high evidence)	RINT1	3 reviews 2 green	BIALLELIC, autosomal or pseudoautosomal	**Sources**<br>• Expert Review Green<br>• Literature<br>**Phenotypes**<br>• Infantile liver failure syndrome 3 OMIM:618641<br>• infantile liver failure syndrome 3 MONDO:0032844<br>**Tags**
GreenGreen List (high evidence)	SERPINA1	5 reviews 3 green	BIALLELIC, autosomal or pseudoautosomal	**Sources**<br>• Expert Review Green<br>• NHS GMS<br>• Other<br>**Phenotypes**<br>• Alpha-1 Antitrypsin Deficiency<br>• Neonatal and Adult Cholestasis<br>**Tags**
GreenGreen List (high evidence)	SLC25A13	4 reviews 3 green	BIALLELIC, autosomal or pseudoautosomal	**Sources**<br>• Expert Review Green<br>• NHS GMS

List	Entity	Reviews	Mode of inheritance	Details

Filter Entities

78 Entities

List	Entity	Reviews	Mode of inheritance	Details
				• Other<br>**Phenotypes**<br>• CHOLESTASIS, NEONATAL INTRAHEPATIC, CAUSED BY CITRIN DEFICIENCY<br>• NICCD<br>• Citrullinemia type 2, neonatal onset<br>• Citrullinemia type 2, adult onset<br>• Citrullinemia, adult-onset type II 603471<br>• Citrullinemia, type II, neonatal-onset 605814<br>• Neonatal and Adult Cholestasis<br>**Tags**
GreenGreen List (high evidence)	SMPD1	2 reviews 1 green	BIALLELIC, autosomal or pseudoautosomal	**Sources**<br>• Expert list<br>• Expert Review Green<br>**Phenotypes**<br>• Niemann-Pick disease, type A, OMIM:257200, MONDO:0009756<br>**Tags**
GreenGreen List (high evidence)	TALDO1	4 reviews 1 green	BIALLELIC, autosomal or pseudoautosomal	**Sources**<br>• Expert Review Green<br>• NHS GMS

List	Entity	Reviews	Mode of inheritance	Details
**Filter Entities** 78 Entities				
				• Other<br>**Phenotypes**<br>• Transaldolase deficiency, 606003<br>**Tags**
GreenGreen List (high evidence)	**TJP2**	3 reviews 3 green	BIALLELIC, autosomal or pseudoautosomal	**Sources**<br>• Expert Review Green<br>• NHS GMS<br>• Other<br>**Phenotypes**<br>• Cholestasis, Progressive Familial Intrahepatic 4<br>• Neonatal and Adult Cholestasis<br>• Cholestasis, progressive familial intrahepatic 4, 615878<br>**Tags**
GreenGreen List (high evidence)	**TRMU**	2 reviews 1 green	BIALLELIC, autosomal or pseudoautosomal	**Sources**<br>• Expert list<br>• Expert Review Green<br>**Phenotypes**<br>• Liver failure, transient infantile, OMIM:613070<br>**Tags**

List	Entity	Reviews	Mode of inheritance	Details
**Filter Entities**				
78 Entities				
GreenGreen List (high evidence)	UGT1A1	3 reviews 2 green	BIALLELIC, autosomal or pseudoautosomal	**Sources**<br>• Expert Review Green<br>• NHS GMS<br>• Other<br>**Phenotypes**<br>• [Gilbert syndrome] 143500<br>• Crigler-Najjar syndrome, type I 218800<br>• Neonatal and Adult Cholestasis<br>• Crigler-Najjar syndrome, type II 606785<br>• unconjugated jaundice<br>**Tags**
GreenGreen List (high evidence)	UNC45A	2 reviews 1 green	BIALLELIC, autosomal or pseudoautosomal	**Sources**<br>• Expert list<br>• Expert Review Green<br>**Phenotypes**<br>• Cholestasis<br>• Diarrhoea<br>• Bone fragility<br>• Impaired hearing<br>**Tags**
GreenGreen List (high evidence)	USP53	3 reviews 2 green 1 red	BIALLELIC, autosomal or pseudoautosomal	**Sources**<br>• Expert Review Green<br>• NHS GMS

List	Entity	Reviews	Mode of inheritance	Details
**Filter Entities** 78 Entities				
				**Phenotypes**<br>• Paediatric cholestatic liver disease<br>• Cholestasis<br>• deafness<br>**Tags**
GreenGreen List (high evidence)	VIPAS39	3 reviews 3 green	BIALLELIC, autosomal or pseudoautosomal	**Sources**<br>• Expert Review Green<br>• NHS GMS<br>• Other<br>**Phenotypes**<br>• Arthrogryposis, Renal Dysfunction, and Cholestasis 2<br>• ARC syndrome<br>• Arthrogryposis-renal-cholestasis syndrome<br>• Neonatal and Adult Cholestasis<br>• Arthrogryposis, renal dysfunction, and cholestasis 2, 613404<br>**Tags**
GreenGreen List (high evidence)	VPS33B	4 reviews 3 green	BIALLELIC, autosomal or pseudoautosomal	**Sources**<br>• Expert Review Green<br>• NHS GMS<br>• Other<br>**Phenotypes**<br>• arthrogryposis-renal-cholestasis syndrome

List	Entity	Reviews	Mode of inheritance	Details
**Filter Entities**				
78 Entities				
				• Arthrogryposis, renal dysfunction, and cholestasis 1, 208085<br>• Arthrogryposis, Renal Dysfunction, and Cholestasis 1<br>• Arthrogryposis, Renal Dysfunction, and Cholestasis Syndrome<br>• ARC syndrome<br>• Neonatal and Adult Cholestasis<br>• Arthrogryposis, Renal Dysfunction, And Cholestasis 1<br><br>**Tags**
GreenGreen List (high evidence)	YARS	3 reviews<br>1 green	BIALLELIC, autosomal or pseudoautosomal	**Sources**<br>• Expert Review Green<br>• Literature<br><br>**Phenotypes**<br>• Charcot-Marie-Tooth disease, dominant intermediate C 608323<br>• Intellectual disability<br>• deafness<br>• nystagmus<br>• liver dysfunction<br><br>**Tags**<br>• new-gene-name
GreenGreen List (high evidence)	ZFYVE19	3 reviews	BIALLELIC, autosomal or pseudoautosomal	**Sources**<br>• Expert Review Green

List	Entity	Reviews	Mode of inheritance	Details
**Filter Entities**				
78 Entities				
		2 green		• Literature<br>**Phenotypes**<br>• Cholestasis, progressive familial intrahepatic, 9, OMIM:619849<br>**Tags**<br>• gene-checked
AmberAmber List (moderate evidence)	FARSA	2 reviews<br>1 red	BIALLELIC, autosomal or pseudoautosomal	**Sources**<br>• Expert Review Amber<br>• Literature<br>**Phenotypes**<br>• ?Rajab interstitial lung disease with brain calcifications 2, OMIM:619013<br>**Tags**<br>• watchlist
AmberAmber List (moderate evidence)	FARSB	1 review	BIALLELIC, autosomal or pseudoautosomal	**Sources**<br>• Expert Review Amber<br>• Literature<br>**Phenotypes**

List	Entity	Reviews	Mode of inheritance	Details
Filter Entities<br>78 Entities				
				• Rajab interstitial lung disease with brain calcifications, 613658<br>**Tags**
AmberAmber List (moderate evidence)	GNAS	3 reviews 1 green	MONOALLELIC, autosomal or pseudoautosomal, NOT imprinted	**Sources**<br>• Expert list<br>• Expert Review Amber<br>• NHS GMS<br>• Other<br>**Phenotypes**<br>• McCune-Albright syndrome<br>• Cholestasis<br>**Tags**
AmberAmber List (moderate evidence)	IARS	2 reviews	BIALLELIC, autosomal or pseudoautosomal	**Sources**<br>• Expert list<br>• Expert Review Amber<br>**Phenotypes**<br>• Growth retardation, impaired intellectual development, hypotonia, and hepatopathy, 617093<br>**Tags**<br>• new-gene-name

List	Entity	Reviews	Mode of inheritance	Details
**Filter Entities** 78 Entities				
AmberAmber List (moderate evidence)	LSR	2 reviews	BIALLELIC, autosomal or pseudoautosomal	**Sources** • Expert Review Amber • Literature **Phenotypes** • transient neonatal cholestasis • intellectual disability • short stature **Tags** • watchlist
AmberAmber List (moderate evidence)	MMP15	2 reviews 1 green	BIALLELIC, autosomal or pseudoautosomal	**Sources** • Expert Review Amber • Literature **Phenotypes** • Cholestasis, MONDO:0001751 • congenital heart disease, MONDO:0005453 **Tags** • watchlist
AmberAmber List (moderate evidence)	NPHP3	2 reviews 1 green	BIALLELIC, autosomal or pseudoautosomal	**Sources** • Expert list • Expert Review Amber

List	Entity	Reviews	Mode of inheritance	Details
**Filter Entities** 78 Entities				
				**Phenotypes** • Renal-hepatic-pancreatic dysplasia 1, 208540 **Tags**
Amber Amber List (moderate evidence)	PEX14	2 reviews	BIALLELIC, autosomal or pseudoautosomal	**Sources** • Expert list • Expert Review Amber **Phenotypes** • Peroxisome biogenesis disorder 13A (Zellweger), 614887 **Tags**
Amber Amber List (moderate evidence)	PEX2	3 reviews	BIALLELIC, autosomal or pseudoautosomal	**Sources** • Expert Review Amber • NHS GMS • Other **Phenotypes** • Neonatal and Adult Cholestasis • Peroxisome Biogenesis Disorder 5A (Zellweger), 614866 **Tags**
Amber Amber List (moderate evidence)	VPS50	2 reviews	BIALLELIC, autosomal or pseudoautosomal	**Sources** • Expert Review Amber

List	Entity	Reviews	Mode of inheritance	Details
**Filter Entities** 78 Entities				
				• Literature<br>**Phenotypes**<br>• Neonatal cholestatic liver disease<br>• Failure to thrive<br>• Profound global developmental delay<br>• Postnatal microcephaly<br>• Seizures<br>• Abnormality of the corpus callosum<br>**Tags**<br>• watchlist
RedRed List (low evidence)	AP1S1	2 reviews 1 green 1 red	BIALLELIC, autosomal or pseudoautosomal	**Sources**<br>• Expert Review Red<br>• Literature<br>**Phenotypes**<br>• Non-syndromic congenital intestinal failure<br>• MEDNIK syndrome, OMIM:609313<br>**Tags**
RedRed List (low evidence)	PPM1F	2 reviews 1 red	BIALLELIC, autosomal or pseudoautosomal	**Sources**<br>• Expert Review Red<br>• Literature<br>**Phenotypes**

List	Entity	Reviews	Mode of inheritance	Details
**Filter Entities**				
78 Entities				
				• sclerosing cholangitis<br>• short stature<br>• hypothyroidism<br>• abnormal tongue pigmentation<br>**Tags**
RedRed List (low evidence)	SLC51A	2 reviews<br>1 red	BIALLELIC, autosomal or pseudoautosomal	**Sources**<br>• Expert Review Red<br>• Literature<br>**Phenotypes**<br>• ?Cholestasis, progressive familial intrahepatic, 6, OMIM:619484<br>**Tags**
RedRed List (low evidence)	WDR83OS	2 reviews<br>1 red	BIALLELIC, autosomal or pseudoautosomal	**Sources**<br>• Expert Review Red<br>• Literature<br>**Phenotypes**<br>• Cholestasis<br>**Tags**
No listNo list	CC2D2A	5 reviews<br>1 green 2 red	BIALLELIC, autosomal or pseudoautosomal	**Sources**<br>• Expert Review Removed<br>• NHS GMS<br>• Other

List	Entity	Reviews	Mode of inheritance	Details
**Filter Entities** 78 Entities				
				**Phenotypes**<br>• COACH syndrome 216360<br>• Meckel syndrome 6 612284<br>• Joubert syndrome 9 612285<br>• Congenital hepatic fibrosis<br>• Ciliopathy<br>**Tags**<br>• curated_removed
No listNo list	RPGRIP1L	4 reviews 1 green 2 red	BIALLELIC, autosomal or pseudoautosomal	**Sources**<br>• Emory Genetics Laboratory<br>• Expert list<br>• Expert Review Removed<br>• Illumina TruGenome Clinical Sequencing Services<br>• NHS GMS<br>• Radboud University Medical Center, Nijmegen<br>• UKGTN<br>**Phenotypes**<br>• Meckel syndrome 5 (611561)<br>• Joubert syndrome 7 (611560)<br>• COACH syndrome (216360)

List	Entity	Reviews	Mode of inheritance	Details
**Filter Entities**<br>78 Entities				
				• Congenital hepatic fibrosis<br>**Tags**<br>• curated_removed
No listNo list	TMEM67	4 reviews<br>2 green 2 red	BIALLELIC, autosomal or pseudoautosomal	**Sources**<br>• Emory Genetics Laboratory<br>• Expert list<br>• Expert Review Removed<br>• Illumina TruGenome Clinical Sequencing Services<br>• NHS GMS<br>• Radboud University Medical Center, Nijmegen<br>• UKGTN<br>**Phenotypes**<br>• COACH syndrome (216360)<br>• {Bardet-Biedl syndrome 14, modifier of} (615991)<br>• Nephronophthisis 11 (613550)<br>• Meckel syndrome 3 (607361)<br>• Joubert syndrome 6 (310688)<br>• congenital hepatic fibrosis<br>**Tags**

List	Entity	Reviews	Mode of inheritance	Details

Filter Entities

78 Entities

				• curated_removed
